# Organization measures in the Enhanced Baking Tray Task

**DOI:** 10.3389/fpsyg.2022.1039064

**Published:** 2022-12-01

**Authors:** Antonietta Argiuolo, Federica Somma, Paolo Bartolomeo, Onofrio Gigliotta, Michela Ponticorvo

**Affiliations:** ^1^Natural and Artificial Cognition Laboratory Orazio Miglino, Department of Humanistic Studies, University of Naples Federico II, Naples, Italy; ^2^CNRS, AP-HP, Hôpital de la Pitié-Salpêtrière, INSERM, Sorbonne Université, Paris, France; ^3^CNRS, AP-HP, Hôpital de la Pitié-Salpêtrière, Institut du Cerveau - Paris Brain Institute - ICM, Sorbonne Université, Paris, France

**Keywords:** E-Baking Tray Task, Baking Tray Task, organization, spatial cognition, visual attention

## Abstract

**Introduction:**

The ecological assessment and the analysis of spatial organization behaviors, like the organization of objects in an empty space, in clinical and neurotypical conditions, is crucial. The Enhanced-Baking Tray Task (E-BTT) is as simple as that – placing objects inside a frame as evenly as possible, as if they were “cookies” to be baked in the oven. The E-BTT is the enhanced version of a task for neglect assessment, the Baking Tray Task, and has the advantage to register the coordinates of each object and their temporal order, meaning that it is easy to reconstruct the sequence of their placement. This sequence could be further analyzed, and, in this paper, we aim to do that with a series of indexes. Moreover, since they investigate the visual search organization of the sequence itself, their validity will be tested with a convergent measure of subjective organization.

**Methods:**

Therefore, we asked 100 observers (76 women) to evaluate the subjective organization of each of 97 E-BTT plots, on a scale that ranged from 0 = not at all to 100 = well organized.

**Results:**

A multiple regression model showed a significant association between subjective organization ratings (dependent variable) and Intersection rate, Total time of performance and distance to both optimal sequences (independent variables).

**Discussion:**

Therefore the above-mentioned indexes can be considered measures of the overall organization in the E-BTT.

## Introduction

Being able to locate salient stimuli in the visual field and paying attention to them is crucial to act in the world. Thus, the way visual search is organized is a key factor both in simple and complex daily life activities, such as arranging objects on a table or driving. Patients with left visual neglect, after right hemisphere lesions, show asymmetrical and inefficient visual search (Bartolomeo, 2014). For example, when asked to cancel multiple targets arranged in space, neglected patients typically omit to cancel left-sided targets. Their path of spatial exploration can be considered a broken line that links each marked target to the other. Whereas neurotypical subjects follow a more ordered and regular cancelation path, patients tend to adopt irregular exploratory patterns (Weintraub and Mesulam, 1988; Gauthier et al., 1989; Donnelly et al., 1999; Woods and Mark, 2007; Warren et al., 2008).

Based on this evidence, Mark et al. (2004) developed three indexes to quantify the spatial organization in a star cancelation task on patients with stroke:

• *Marking distance*, the Euclidean distance between one canceled target and the next one; an organized pattern means minimizing the distances between the targets, corresponding to shorter marking distances.
• *The number of intersections* in the reconstructed cancelation pattern. It was divided by the total number of marks for different targets (i.e., not considering perseverations); in an organized path, a lower rate of intersections was expected.
• *Best r*, the highest Pearson’s coefficient between the correlation of the X-coordinates with the relative order and the correlation of the Y-coordinates with the relative order, computed separately. Healthy subjects explore the targets with a horizontal or vertical path, and this index thus manages to capture this orthogonal movement. Therefore, the higher Best r is, the more organized the pattern should correspond.

To test the validity of these indexes, Woods and Mark (2007) administered to 50 adult observers 50 plots that reconstructed just as many pathways in a cancelation task. Participants assessed on a scale from 0 (= most organized) to 10 (= least organized) the overall organization of each path. All three Mark’s indexes (marking distance, Best r, and number of intersections) were found to be associated with observers’ scores or subjective organization.

Other authors tried to quantify the visual organization in cancelation tasks using these indexes (Ten Brink et al., 2016a,b, 2018; Benjamins et al., 2019) or tried to develop other measurements (Dalmaijer et al., 2015). Among them, it is worth mentioning

• *Total time* of the performance.
• *Search speed*, the mean of the ratio between each inter-cancelation distance and each inter-cancelation time.
• *Standardized angle*, which integrated Mark et al. (2004) Best r. If the Best r captures the orthogonal patterns, the standardized angle allows considering irregular (non-horizontal or non-vertical) patterns, too.

**FIGURE 1:**
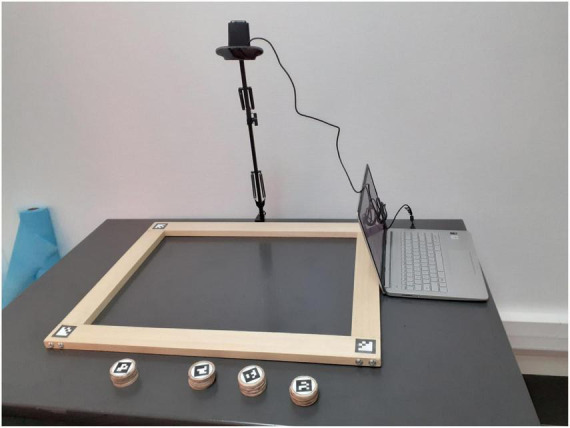
The Enhanced Baking Tray Task (E-BTT) apparatus.

These and other indexes were applied in a recent study (Argiuolo et al., 2022), whose aim was to generalize these organization indexes to a new tool, the Enhanced Baking Tray Task (E-BTT). The E-BTT (Cerrato et al., 2019a,b, 2020; Gentile et al., 2019; Palumbo et al., 2019; Argiuolo and Ponticorvo, 2020; Somma et al., 2020; Argiuolo et al., 2021a) exploits tangible interfaces and has been developed based on the baking tray task, a widely used neglect assessment tool (Tham and Tegnér, 1996). The E-BTT is made up of 16 disks and a modular wooden frame (60 cm × 45 cm) equipped with tags (ArUco Markers, Garrido-Jurado et al., 2014), which are detected by a camera placed above the table, attached to a metallic arm (refer to Figure 1). Participants should place the disks, one by one, inside the frame as evenly as possible “as they were cookies to be baked in the oven.” The order is not predefined, but once placed, the disk can no longer be moved. A software platform, called E-TAN, processes the positions of the disks and returns a set of 16 ordered coordinates as output (Cerrato et al., 2020).

The fundamental difference with the cancelation task is the absence of predefined and therefore correct answers: the disks can be placed potentially anywhere and in any order, not decided *a priori*. This difference may trigger different spatial processes: if in the cancelation, it is essential to pay attention to the targets (and discriminate them from the distractors); in the E-BTT, each participant has an empty space in front of them in which they have to organize and fill in with the disks, knowing that they cannot be moved once they are placed. Moreover, the E-BTT can be considered ecologically valid, inasmuch as it refers to an everyday activity such as disposing of objects, and has a somatic component since it requires physically grabbing each disk and placing it inside the frame. It is more and more clear the importance of preserving the interaction with tangible objects in neuropsychological assessment. More awareness of the resulting bodily and situated aspects of spatial behaviors is needed (Pfeifer et al., 2007; Clark, 2008).

However, despite the differences, the organization of visual exploration should be crucial to process the space in the E-BTT, too. Indeed, the E-BTT needs the participant to organize the framed space, using it as much as possible and using all the available disks. Also, the sequence that goes from the first to the last-placed disk can be considered, which will result in a broken line just like in cancelation tasks pathways.

The abovementioned indexes (inter-disks distance, intersection rate, etc.) were proven to be usefully applied to the E-BTT (Argiuolo et al., 2022). Moreover, their structure was studied through a principal component analysis, and three out of the five latent components that emerged could be linked to the quality of the sequence itself. These latter indexes were then used in this article, starting from the hypothesis that they measure the organization/disorganization of the spatial configuration of the disks in the E-BTT. In other words, we expect that these indexes are significantly associated with the subjective judgment of external observers in terms of organization/disorganization, as Woods and Mark (2007) have already noted for the cancelation test.

## Materials and methods

### Participants

A total of 208 healthy participants responded to a Qualtrics^®^ online survey, but only 100 of them completed it. Further analyses were drawn on these 100 observers. They identified as women (*N* = 76), men (*N* = 22), or non-binary (*N* = 2). Their mean age was 29.94 years (SD = 7.59). Approximately 46% of them studied or worked in the psychology field. Informed consent was obtained from all participants. The present study was approved by the University of Naples “Federico II” Local Ethics Committee (protocol number: 21/2021) and was conducted in accordance with the Declaration of Helsinki.

### Measures

#### Enhanced Baking Tray Task plots

**FIGURE 2:**
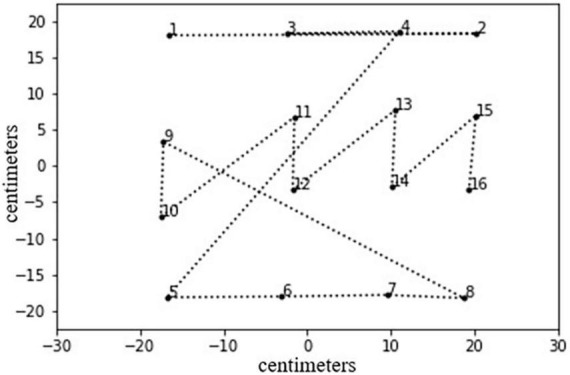
An example of an E-BTT plot as used in the online survey. Black numbered dots represent each disk and the dashed line is the sequence.

Participants were asked to look carefully at 97 E-BTT plots and judge them for their overall organization. Data used for the E-BTT plots in this article were published by Somma et al. (2020). In Figure 2, there is an example of an E-BTT plot. Black dots represent the disks; they were numbered so that each participant could follow the sequence (drawn with a dashed line) that goes from the first to the last disk. They referred to 97 (78 women) healthy participants, whose ages ranged from 18 to 26 years (mean = 20; SD = 1.44), 7 of them were left-handed (for more details, refer to Somma et al., 2020).

It is true that we chose to use healthy subjects’ plots, and this could affect the results so that they could have followed homogeneous strategies and organized sequences may be overrepresented. However, the E-BTT plots data set can be considered adequate because they were sufficiently heterogenous and the disorganized sequences were frequent. As a matter of fact, in a recent study (Argiuolo et al., 2021b) they were analyzed through Euclidean distances. Eleven groups of strategies emerged, and they did not explain all the samples; moreover, the sample was subjectively divided into “organized” and “disorganized” sequences and their numerosity was, respectively 53 and 44.

#### Organization indexes

**FIGURE 3:**
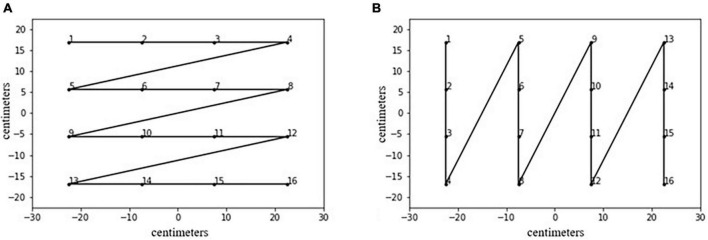
The two optimal sequences used. The first is a sawtooth by rows **(A)**; the second is a sawtooth by columns **(B)**.

A series of indexes were calculated for the 97 available sequences:

• *Inter-disks distance* (within distance, WD), an index similar to that from Mark et al. (2004): The sum of the Euclidean distance within each disk and the next one. We expected that the WD would be minimized in organized plots.
• *Intersection rate*, the number of intersections divided by the total number of segments (15): An intersection in the reconstructed path could mean a sort of revisiting of already explored space; a well-organized path should minimize revisiting and thus intersections.
• *R_X and R_Y*: Unlike Mark et al. (2004) or Dalmaijer et al. (2015), we decided to use both correlations of the coordinates and their order and not only the highest between the two. Thus, they were considered separately in further analysis. Our objective was to obtain a more refined way of reconstructing the path: a high absolute value of R_X indicates a mainly vertical path; a high absolute value of R_Y indicates a mainly horizontal path. We expected that both indexes would correlate with the subjective organization because a clearly orthogonal path should be judged as more organized.
• *Total time*, measured in seconds, of the E-BTT performance: Having in mind a plan on how to place the disks should result in faster and better organized performance.
• *Global speed*: Unlike Dalmaijer et al. (2015), it was calculated by dividing the total WD by the total time. It is expected that a higher speed should result in a more organized path.
• *Standardized angle*, calculated as in Dalmaijer et al. (2015), to integrate the Best r measures: It consisted of the arcsin of the ratio between the vertical distance between two points and their Euclidean distance. According to Dalmaijer et al. (2015), higher standardized angles should reflect more organized sequences.
• *Total area*, a measure used by Cerrato et al. (2020) consisting of the proportion of the explored area: It was calculated as the convex hull polygon delimited by the disks’ sequence [for more details, refer to Cerrato et al. (2020)]. A well-organized sequence in our case should result in an optimal portion of explored space.
• *Optimal sequences between distance* (BD), the Euclidean distance between each sequence and two optimal sequences: These (refer to Figure 3) were created by dividing the available space into 16 equal parts and calculating the coordinates as the disks were placed in the center of each of these parts. The two sequences were chosen based on the fact that a sequence that goes by rows or columns was considered well organized, and they were actually more frequent (Argiuolo et al., 2021b). In our hypothesis, given that a path by row and columns can be considered organized, the more distant a sequence from these optimums, the more disorganized it would be considered.

#### Subjective organization

A measure of subjective organization was implemented. We asked the participants to assess, for each plot, the overall organization on a continuous scale from 0 = not at all organized to 100 = well organized.

#### Procedure

A Qualtrics^®^ link was generated and spread through social media. After a few demographics (age, gender, handedness, last study level, occupation, and if worked in/studied psychology), the E-BTT task was described to the participant (without mentioning the indexes). There were three trial plots to allow the observer to feel confident about the task. The three plots used for the trial tests were not registered. After the trial, the real observation started, and it was structured as follows: a plot was displayed with the scale below it; the observer had to move the cursor from 0 to the desired number. The order of the plots was randomized. In the end, there was a facultative question about how each of them interpreted the concept of the organization during the task. The overall procedure took about 30 min.

## Results

Due to non-normal distributions, non-parametric analyses were preferred. The subjective organization measure’s median for each item (plot) was used in Spearman’s ρ to evaluate convergence. Table 1 summarizes the most important descriptive statistics for each index and the subjective organization measure.

**TABLE 1:** Descriptive statistics for the indexes and the measure of subjective organization.

		Subjective organization	WD	Intersection rate	R_X	R_Y	Total time	Global speed	Standardized angle	Total area	OS1_BD	OS2_BD
Mean		53.13	255.44	0.19	0.337	–0.08	59.62	5.09	1	0.35	357.6	356.57
Median		52.00	240.50	0	0.307	–0.13	49	4.67	1	0.37	393.77	378.09
SD		24.56	90.71	0.41	0.495	0.65	44.85	2.17	0.01	0.14	135.24	124.12
Asymmetry		0.07	0.77	2.71	–0.749	0.12	5.61	0.25	0.08	–0.39	–1.2	–0.83
Kurtosis		–1.21	0.89	7.16	0.582	–1.16	39.9	–0.38	0.82	–0.11	0.66	0.99
Min		10	83.18	0	–0.971	–0.98	24	0.52	0.98	0.04	23.19	33.11
Max		96	516.33	1.93	0.982	0.98	408	11.32	1.02	0.64	544.38	635.12
Percentiles	25	31	205.39	0	0.095	–0.69	39	3.35	0.99	0.29	312.88	321.45
	75	74	296.22	0.13	0.78	0.433	69	6.6	1	0.43	446.29	418.76

OS1_BD, between distance from optimal sequence number 1 (a sequence characterized by a sawtooth by rows); OS2_BD, between distance from optimal sequence number 2 (a sequence characterized by a sawtooth by columns).

The Spearman’s ρ correlations (Table 2) showed a significant association between subjective organization ratings and Intersection rate (*r* = –0.77, *P* < 0.05), correlations of each coordinates series and their order (respectively R_X: *r* = 0.26, *P* < 0.05 and R_Y: *r* = –0.36, *P* < 0.05), total time of performance (*r* = –0.3, *P* < 0.05), and distance to both optimal sequences (*r* = –0.36, *P* < 0.05 and *r* = –0.33, *P* < 0.05).

**TABLE 2:** Spearman’s ρ correlation between the indexes and the subjective organization.

	Subjective organization	WD	Intersection rate	R_X	R_Y	Total time	Global speed	Standardized angle	Total area	OS1_BD	OS2_BD
Subjective organization	1.000	−0.19	−0.77*	0.26*	−0.36*	−0.3*	0.075	0.19	−0.04	−0.36*	−0.33*

OS1_BD, between distance from optimal sequence number 1 (a sequence characterized by a sawtooth by rows); OS2_BD, between distance from optimal sequence number 2 (a sequence characterized by a sawtooth by columns).

*p < 0.05.

Bold values represent the significant values.

In order to analyze the eventual collinearity between these indexes, we also performed a correlation analysis (Table 3). All significant correlations were below 0.4, except the correlation between R_X and OS2_BD and the correlation between R_Y and OS1_BD.

**TABLE 3:** Spearman’s ρ correlation between the indexes.

	Intersection rate	R_X	R_Y	Total time	OS1_BD	OS2_BD
Intersection rate	–					
R_X	−0.329*	–				
R_Y	0.136	0.037	–			
Total time	0.381*	−0.068	−0.048	–		
OS1_BD	0.184	−0.042	0.799*	0.025	–	
OS2_BD	0.289*	−0.808*	0.314*	0.063	0.311*	–

OS1_BD, between distance from optimal sequence number 1; OS2_BD, between distance from optimal sequence number 2.

*p < 0.05.

Bold values represent the significant values.

We then inserted these indexes in a multiple regression model in order to obtain the relation between each of them and the subjective organization. Results showed that intersection rate, total time of performance, and distance to both optimal sequences are significantly related to the subjective evaluation of the organization, while R_X and R_Y’s estimates were not statistically significant (Table 4). The model explained about 48% of the variance (adjusted *R*^2^ = 0.48) and the root mean square error was equal to 17.09. The collinearity statistics (Table 5) showed that there was no concern about collinearity because all variance inflation factor coefficients are below 5.

**TABLE 4:** Multiple regression analysis of the indexes on the subjective evaluation of the organization.

						95% confidence interval	
Predictor	Estimate	SE	*t*	*p*	Stand. estimate	Lower	Upper
**Model coefficients – Subjective_organization**							
Intercept	107.420	15.122	7.104	<0.001*			
Intersection rate	−26.309	4.867	−5.406	<0.001*	−0.436	−0.596	−0.275
R_X	−7.168	6.596	−1.087	0.280	−0.144	−0.408	0.120
R_Y	−2.367	4.525	−0.523	0.602	−0.062	−0.299	0.174
Total time	−0.095	0.043	−2.226	0.029*	−0.174	−0.329	−0.019
OS1_BD	−0.050	0.021	−2.430	0.017*	−0.277	−0.503	−0.050
OS2_BD	−0.066	0.027	−2.429	0.017*	−0.332	−0.604	−0.060

*p < 0.05.

**TABLE 5:** Collinearity statistics.

	VIF	Tolerance
**Collinearity statistics**		
Intersection rate	1.194	0.838
R_X	3.246	0.308
R_Y	2.603	0.384
Total time	1.119	0.893
OS1_BD	2.382	0.420
OS2_BD	3.437	0.291

**FIGURE 4:**
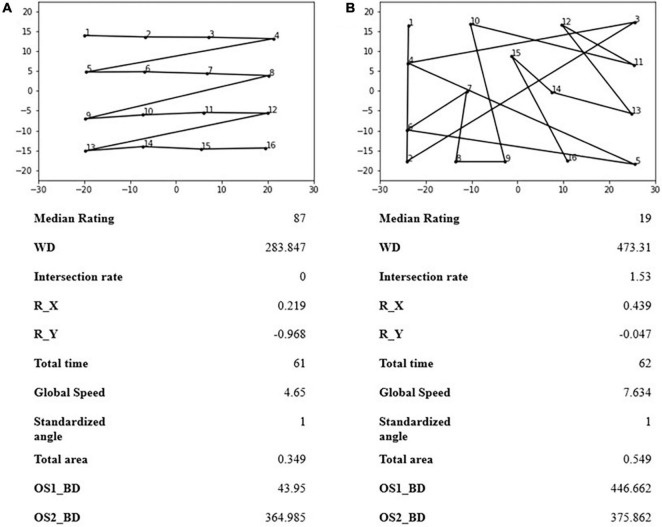
Two examples of E-BTT sequences, one **(A)** evaluated as organized and the other **(B)** with low subjective ratings of organization. OS1_BD, between distance from optimal sequence number 1 (a sequence characterized by a sawtooth by rows); OS2_BD, between distance from optimal sequence number 2 (a sequence characterized by a sawtooth by columns).

In Figure 4, two example sequences are shown. These two examples were chosen based on their subjective overall ratings. The first, in Figure 4A, is identified as well organized with high subjective ratings, no intersection, and a low distance from one of the two optimal sequences. The second, instead, has a high intersection rate, low subjective rating, and high distance from both optimal sequences. These can be considered two extremes in the subjective organization–disorganization continuum.

### The concept of “organization”

As the last question, participants were asked to report their concept of “organization” and which criterion they had used to judge the plots. They report they followed “order of the sequence,” “symmetry,” and “sequentiality of numbers” in their evaluation. They also relied on “order,” “symmetry,” “harmony,” and “linearity” of each sequence; some also highlighted they considered organized the plot whose lines did not intersect or overlap. Many also referred to the “homogeneity” of space used.

## Discussion

In this article, we explored how some indexes, recently applied to the E-BTT, could measure the organization of the spatiotemporal sequence output of the task. To do so, a subjective evaluation of each pattern’s overall organization was assessed. No information on the indexes was given to participants, but there was, however, a detailed description of the E-BTT and how it worked in order to let the participants familiarize themselves with the task.

Our initial hypotheses were only partially confirmed by results because not all the indexes included in this study were significantly associated with the subjective evaluation of spatial organization. From these results, it can be concluded that a plot was considered organized if

• it has a low intersection rate;
• the E-BTT performance time was low; and
• the distance from both optimal sequences was short.

The intersection rate correlated negatively with the subjective evaluation scores of the overall organization because a higher number of intersections in the sequence were associated with lower ratings of the organization. The intersection rate has been widely used to measure search organization (Mark et al., 2004; Ten Brink et al., 2016a,b, 2018; Benjamins et al., 2019) and was associated with the activation of posterior regions of the right hemisphere in patients with stroke (Ten Brink et al., 2016a). Thus, disorganized search patterns seem to be associated with brain regions dedicated to conjunctive search and spatial working memory rather than with prefrontal regions (responsible for executive functions).

The amount of time a participant employs to finish a task can be considered a measure of executive functioning (Dalmaijer et al., 2015) because it reflects the ability to use one’s attentive resources throughout the whole task. The fast performance of the E-BTT may depend on a proper plan of action, resulting in an organized sequence of placement. Consistently, performance time was negatively associated with the subjectively judged organization. In other words, the plots judged as more organized were those completed faster.

Interestingly, the correlation between the Y-coordinates and their order is negatively associated with the overall subjective organization, whereas it is positively correlated to R_X. These two results were not confirmed when these two indexes were inserted in a multiple regression model, though. This could be explained by the high correlation between R_X and OS2_BD and between R_Y and OS1_BD which probably made spurious the relationship between R_X, R_Y, and the subjective evaluation.

Optimal sequences between distance is the Euclidean distance from each of the two sequences considered optimal. Our data suggest that the more a sequence is distant from these two sequences, the more disorganized they are evaluated. This could mean that if the sequence is very different from one of the two optima, it can be considered disorganized, thus implying that the two optimal sequences are organized.

In our study, WD (similar to inter-cancelation distance) was not associated with subjective evaluation, unlike in previous studies (Woods and Mark, 2007). This could mean that in E-BTT the intra-disks distance is not an organization index, but rather an index of how much space is used in the task, like the total area (Argiuolo et al., 2022). In our previous study, however, WD could actually discriminate between organized and disorganized plots (Argiuolo et al., 2021b), so further studies are needed to solve this issue.

Speed, standardized angle, and total area were not associated with the evaluation of the plot organization. Thus, perhaps the organization’s judgments did not take into substantial account the amount of space used by the sequence. When asked about their concept of “organization,” many refer to “order of the sequence,” “symmetry,” “sequentiality of numbers,” etc. Indeed, the observers reported having relied on “order,” “symmetry,” and “linearity” to judge each sequence as they report in the last question.

This study is not without limitations. First, we used a subjective measure of the overall organization, with all the limitations that a subjective measure entails (social desirability, response set, fatigue, etc.). In addition, participants were asked to judge the plots for their “overall organization” without a clear operationalization of the construct “organization” and thus relying on everyone’s subjective concept. It is also true that we followed Woods and Mark’s (2007) example, and they did not give participants a clear definition of “organization” either.

Secondly, the measure of subjective organization could be considered a measure of convergent validity, part of the construct validity (Rosenthal and Rosnow, 1991). Discriminant validity was not considered in this paper, so future studies should address discriminant validity in organization indexes in E-BTT.

Moreover, the plots we used referred all to healthy participants (Somma et al., 2020), while Woods and Mark (2007) also used reconstructed paths from patients with stroke. It would be interesting to test whether there were any differences in terms of organizational measures in the E-BTT between patients and healthy participants.

## Conclusion

In conclusion, total time, distance from optimal sequences, and intersection rate can be considered indexes of spatial organization in the E-BTT task. Future studies should deepen the issue, distinguishing different neuropsychological syndromes resulting from different brain lesions. In parallel, the use of these indexes for research on healthy participants could help clear the relationship between spatial abilities and other important psychological variables, such as stress (Somma et al., 2021).

## Data availability statement

The raw data supporting the conclusions of this article will be made available by the authors, without undue reservation.

## Ethics statement

The studies involving human participants were reviewed and approved by the Ethical Committee of Psychological Research, University of Naples Federico II, Department of Humanistic Studies. The patients/participants provided their written informed consent to participate in this study.

## Author contributions

OG, MP, and PB contributed to the conception and design of the study. AA and FS collected the data. AA wrote the first draft and performed the statistical analysis. All authors contributed to the manuscript revision, read, and approved the submitted version.
